# Self-Treatment of Chronic Low Back Pain Based on a Rapid and Objective Sacroiliac Asymmetry Test: A Pilot Study

**DOI:** 10.7759/cureus.19483

**Published:** 2021-11-11

**Authors:** Helene Bertrand, K. Dean Reeves, Rajneet Mattu, Remerlita Garcia, Mahir Mohammed, Ellen Wiebe, An-Lin Cheng

**Affiliations:** 1 Department of Family Practice, University of British Columbia Medical School, North Vancouver, CAN; 2 Rehabilitation Medicine, Independent Researcher, Kansas City, USA; 3 Faculty of Science, University of British Columbia, British Columbia, CAN; 4 Obstetrics and Gynaecology, Independent Researcher, Vancouver, CAN; 5 Family Practice, Independent Researcher, Vancouver, CAN; 6 Department of Family Medicine, University of British Columbia Medical School, Vancouver, CAN; 7 Biomedical and Health Informatics, University of Missouri Kansas City School of Medicine, Kansas City, USA

**Keywords:** treating low back pain, sacroiliac joint manipulation, sacroiliac, sacroiliac joint dysfunctional pain, sacroiliac displacement, low back pain physical exam, acute low back pain, lower back pain (lbp), chronic non-specific low-back pain, chronic low back pain (clbp)

## Abstract

Background: Low back pain (LBP) is common, costly, and disabling. This study assesses a novel and simple LBP evaluation method and its merit in guiding the direction of a self-treatment exercise.

Methods: Randomized open-label intention is used to treat the study. Consecutive patients with LBP ≥ three months and pain ≥ 5/10 were evaluated in a Vancouver clinic with the sacroiliac forward flexion test (SIFFT) by comparing the height of posterior superior iliac spines using a level. Those with asymmetry ≥ 5 mm were offered participation. The assistant, who generated and encrypted the randomization, assigned participants: group 1 learned a two-minute, SIFFT-derived, sacroiliac-leveling exercise (SIFFT-E) as needed for LBP relief; group 2 used a pelvic stabilization belt as needed to prevent LBP, and group 3 continued the usual care. After one month, all participants used SIFFT-E and belt as needed for one month. The identifier number of this article in Clinicaltrials.gov is #NCT03888235. The trial is closed.

Our primary outcome measure was the Oswestry disability index (ODI) (decrease) from baseline to one and two months. We also followed SIFFT improvement (decrease).

Findings: Of 72 LBP patients, 62 (86%) had ≥ 5 mm asymmetry. From zero to one month, the 21 (one dropout) SIFFT-E participants outperformed the 20 usual care participants for ODI improvement (12.5 ± 14.8 vs. -3.4 ± 14.9 points; mean difference 15.9 [CI 6.7-25.0]; P = 0.002 with number needed to treat (NNT) of 3.0 for ODI improvement ≥ 11). Belt use results were intermediate. At two months, after all the 62 participants used the exercise and belt as needed, combined ODI improvements were clinically significant (12.0 ± 18.4 points), and SIFFT asymmetry was reduced by 8.6 ± 8.6 mm. Five (8%) exercise and 12 (19%) belt wearers experienced mild side effects.

Interpretation: Sacroiliac asymmetry appears to be frequent. SIFFT may be clinically useful as an evaluation tool for prescribing a simple self-directed corrective exercise as seen by clinically significant improvements in function and asymmetry.

## Introduction

Low back pain (LBP) is a strong driver of medical visits, expensive treatments, and absenteeism [1-3]. Worldwide, back pain is now the most common cause of disability [4], but most often, the cause is considered nonspecific [5,6]. The sacroiliac joint (SIJ) is a common source of LBP, and yet, LBP is commonly treated generically whether or not the SIJ is a pain generator. Malalignment is difficult to assess with medical imaging [7-9]; also, tests for sacroiliac dysfunction are not reliable and do not indicate therapeutic options [10-14]. Currently, in patients suffering from sacroiliac joint pain, decreased strain on pelvic stabilizer muscles and pain relief have been obtained with a pelvic compression belt [15-17].

A new examination that Dr. Bertrand developed in 2014, the sacroiliac forward flexion test (SIFFT) (see Section "Sacroiliac Forward Flexion Test" and the video "How to diagnose and treat low back pain from sacroiliac joint displacement" given in the section "Group 2: Pelvic Stabilizer Belt"), uses a small level to measure the height difference between left and right posterior superior iliac spines (PSISs) and determine which is higher, indicating the choice of leveling exercise (SIFFT-E). A quality assurance retrospective chart review was conducted on the results from the application of the SIFFT and SIFFT-based exercises on 180 consecutive patients presenting with LBP. At that time, the most frequently recommended corrective exercise required an assistant. A SIFFT asymmetry was found in 164 patients (91%). Of those with asymmetry, 146 found immediate relief with sacroiliac (SI) leveling exercises (101 experienced complete and 45 experienced partial pain relief) (Appendix A). Although the duration of benefit and ability of the patients to reproduce the exercise at home were not followed, these results provided the motivation for initiating this pilot study.

We hypothesize that the use of the SIFFT-E will provide clinical improvement in back function (ODI) and brief pain inventory (BPI) by a reduction in measured innominate torsion in comparison with the usual care group at one month. We expect intermediate results in the group treated with a pelvic compression belt [16]. After all the participants use the as-needed exercise and a pelvic support belt from month one to month two, we hypothesize that all will experience improved ODI, SIFFT, and pain levels compared to those at their initial visit.

## Materials and methods

Study design

This randomized open-label study was carried out in family practice in North Vancouver, Canada. The University of British Columbia Clinical Research Ethics Board approved this study (Approval No.: H19 - 01224). The protocol was found at www.clinicaltrials.gov with the identifier number NCT03888235.

Participants

All new patients scheduled to attend the office for LBP were informed about the study (https://low-back-pain.squarespace.com) and given the consent form online or in their appointment.

Participants between 19 and 90 years of age with LBP (primarily below their waist) for ≥3 months and a brief pain inventory pain severity score (BPI) ≥ 5/10 were included. The distance between their PSIS levels was ≥5 mm, and the long dorsal sacroiliac ligament below at least one of the PSISs was tender to pressure on initial examination. The main inclusion criterion for participation in the study was a distance between the PSIS levels ≥ 5 mm. This excluded other causes of LBP such as local or systemic pathologies, previous surgeries, and visceral dysfunctions of the pelvic floor, which are not associated with sacroiliac joint displacement. Leg length discrepancy > 3 cm, severe systemic illness, primary pain elsewhere, and sciatic neuropathy were the exclusion criteria. In order to reach 20 in each group with data gathered for two months, a decision was made that if a participant could not be reached for five months, he/she would be replaced and their data would be carried forward. The replacement participants were not randomized.

Sacroiliac Forward Flexion Test (SIFFT)

**Figure 1:**
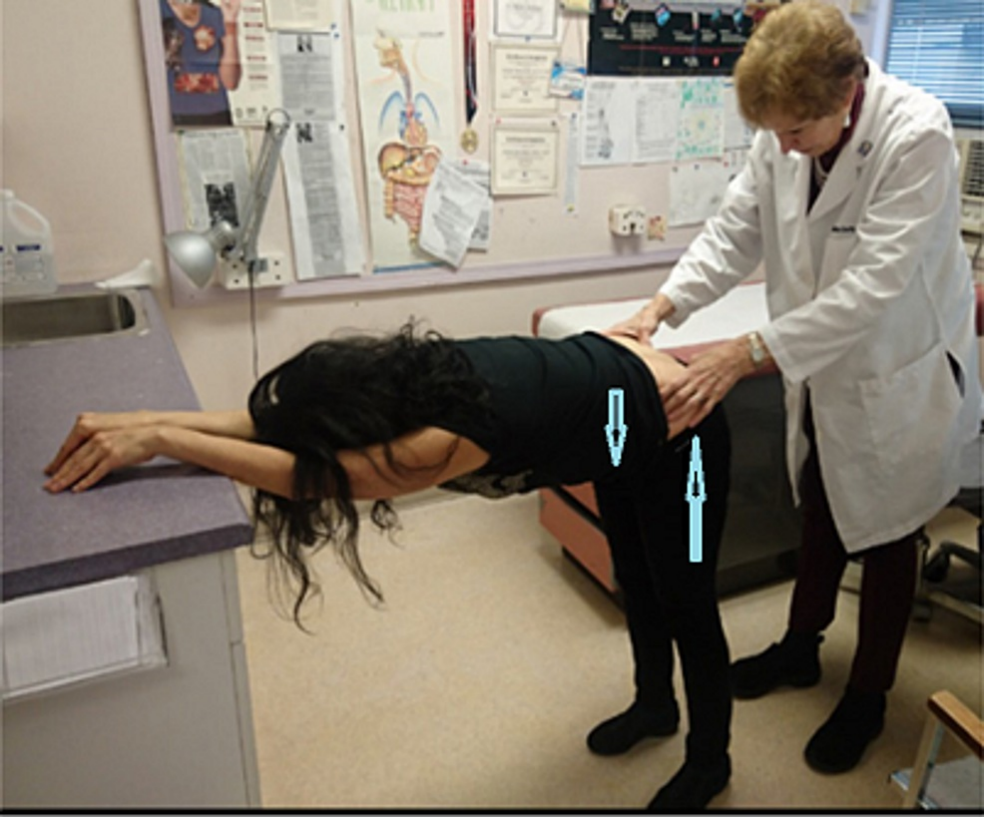
Sacroiliac forward flexion test (SIFFT) The patient is leaning against the desk to relax the buttock muscles, making them softer and easier to press down on. The legs push the PSISs up, while the weight of the body brings the spine down, which rotates the sacrum anteriorly making the PSISs easier to feel. PSISs are located by pressing down firmly with the ulnar side of the thumbs while gradually advancing the cephalad toward the PSISs starting on either side of the intergluteal cleft. When the PSISs are reached, bony resistance is felt. For a video demonstration of the SIFFT technique, please refer to Video 1. The space between the thumb and the underside of each PSIS is marked with a surgical marker. The examiner then asks the participants whether and on which side they feel pain. The examiner then writes the letter B (for before) on the painful side or both sides if they have pain on both sides. In those with obesity or very muscular participants, ultrasound can be used to determine the PSIS levels.

Leg length asymmetry was measured from umbilicus to medial malleolus and equalized to ≤1 cm difference prior to SIFFT by the addition of lifts to the short side. All nonspecific LBP patients stood with their legs vertical, belts undone, and their body flexed at the hips as horizontal as feasible with their arms resting on a surface for the SIFFT test (Figure 1). The examiner, Dr. Helene Bertrand, Dr. Remerlita Garcia, or Dr. Mahir Mohammed, marked the area between their thumb and the bottom edge of the PSIS on each side and wrote the letter B (for before) on the painful side or both sides if they had pain on both sides. Ultrasound can potentially locate the bottom of the PSIS with more precision, but this technology was not used in the present study.

**Figure 2:**
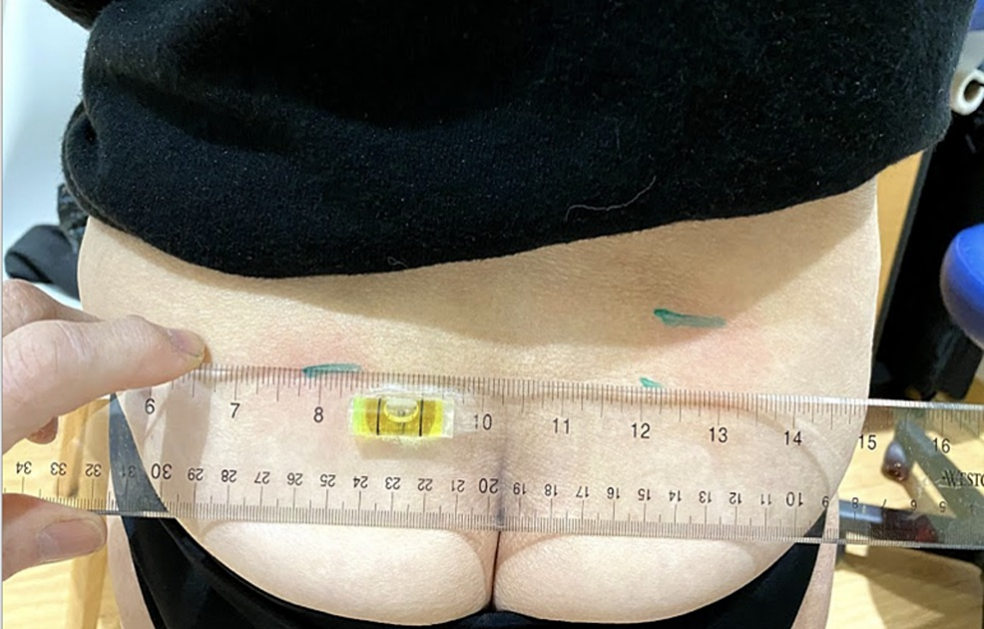
SIFFT: Using a level on the lowest PSIS and marking the corresponding area under the higher PSIS SIFFT, Sacroiliac forward flexion test; PSIS, posterior superior iliac spine.

**Figure 3:**
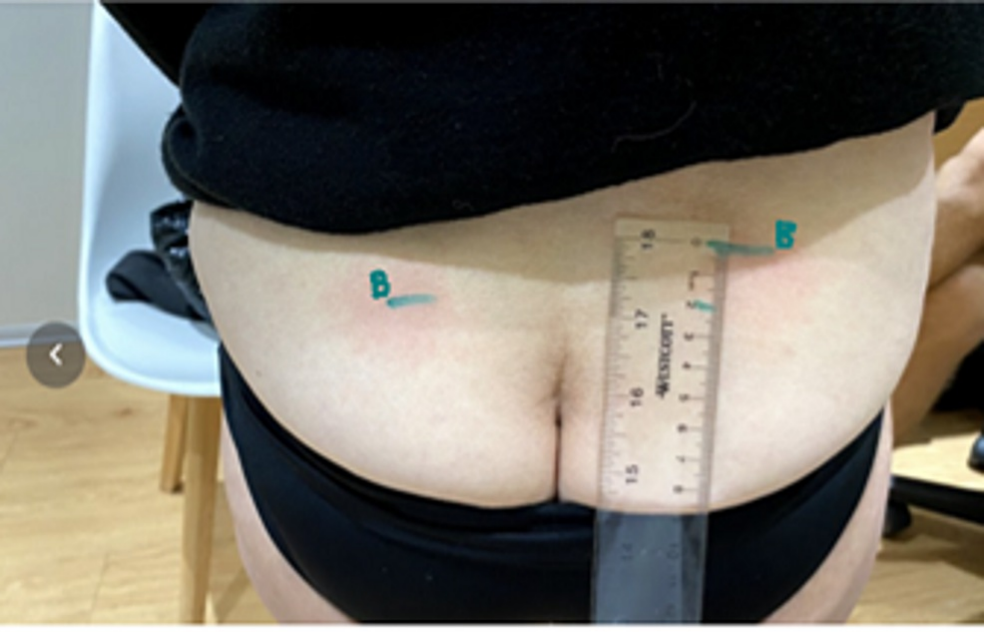
SIFFT: Measuring the distance between PSIS levels in cm SIFFT, Sacroiliac forward flexion test; PSIS, posterior superior iliac spine.

They then asked the patient to stand and place a horizontal level on the lowest mark (Figure 2) and measured the SIFFT: the distance between the level of the lowest and that of the highest mark in millimeters (Figure 3). The work of Levangie [18] established the intra-rater accuracy of a manual PSIS height measurement. She used the horizontal arm of a pedestal-mounted post to measure the height of different pelvic structures. She found that the height of the PSIS measurement was most accurately measurable among pelvic structures with a margin of error of 2.7 mm (intraclass correlation coefficient, ICC = 0.998). Comparing the height of these structures helped determine the presence of sacroiliac torsion with a margin of error of 3.3 mm (ICC = 0.70). She chose 6 mm as the cutoff point to differentiate between subjects with and without sacroiliac torsion. We expected our accuracy to be more precise because the patient was measured in flexion, stretching soft tissue, thus facilitating palpation of the inferior edge of the PSIS. Therefore, we chose 5 mm as indicative of significant sacroiliac torsion. As ground-based height measurement systems are cumbersome and as the important measurement is the difference between the PSIS levels, we used a small level and an ordinary ruler. To compare PSIS heights, both the ruler and level were sanded where they were to be glued and the cyanoacrylate glue was used to bind them together. If the SIFFT was ≥ 5 mm, the patient was randomized to participate in the study.

Randomization

Using Research Randomizer (www.https://www.randomizer.org), 20 sets of three numbers (1, 2, 3) were generated by the medical office assistant and kept in an encrypted file in her password-protected computer. Once participants provided baseline data and were examined and deemed eligible to participate by one of the study physicians, they signed the consent form and were assigned to their treatment group by the assistant, following which she had no further involvement in the study. Those involved with statistical analysis and interpretation had no contact with participants and received de-identified data.

Procedures

Group 1: SIFFT-Guided Leveling Exercise

**Figure 4:**
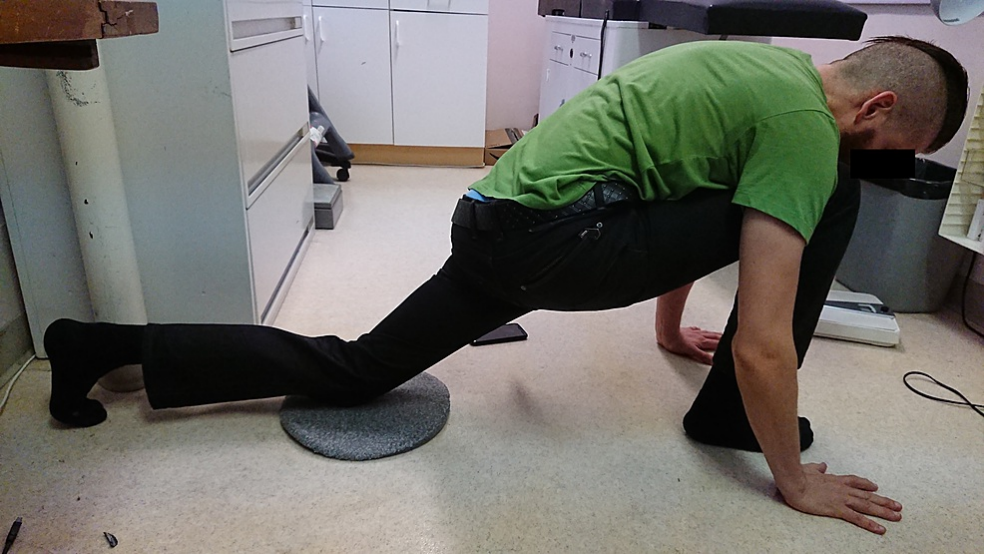
SIFFT-E stretch exercise to level a right anterior, left posterior sacroiliac malrotation Place the right foot and the left knee on the floor hands on either side of the right foot. Lean forward so that the right thigh is pushing up hard on the right ASIS to level an anterior SI torsion. Slide the left knee as far back as possible to hyperextend the left thigh. A strong pull should be felt in the left groin to level a posterior SI torsion. Hold this position for a full two minutes. If only one side is affected, less pull or pressure is exerted on the nonpainful side. Of the 62 participants, 51 did this exercise. SIFFT, Sacroiliac forward flexion test; SIFFT-E, SIFFT-guided leveling exercise; SI, sacroiliac; ASIS, anterior superior iliac spine.

**Figure 5:**
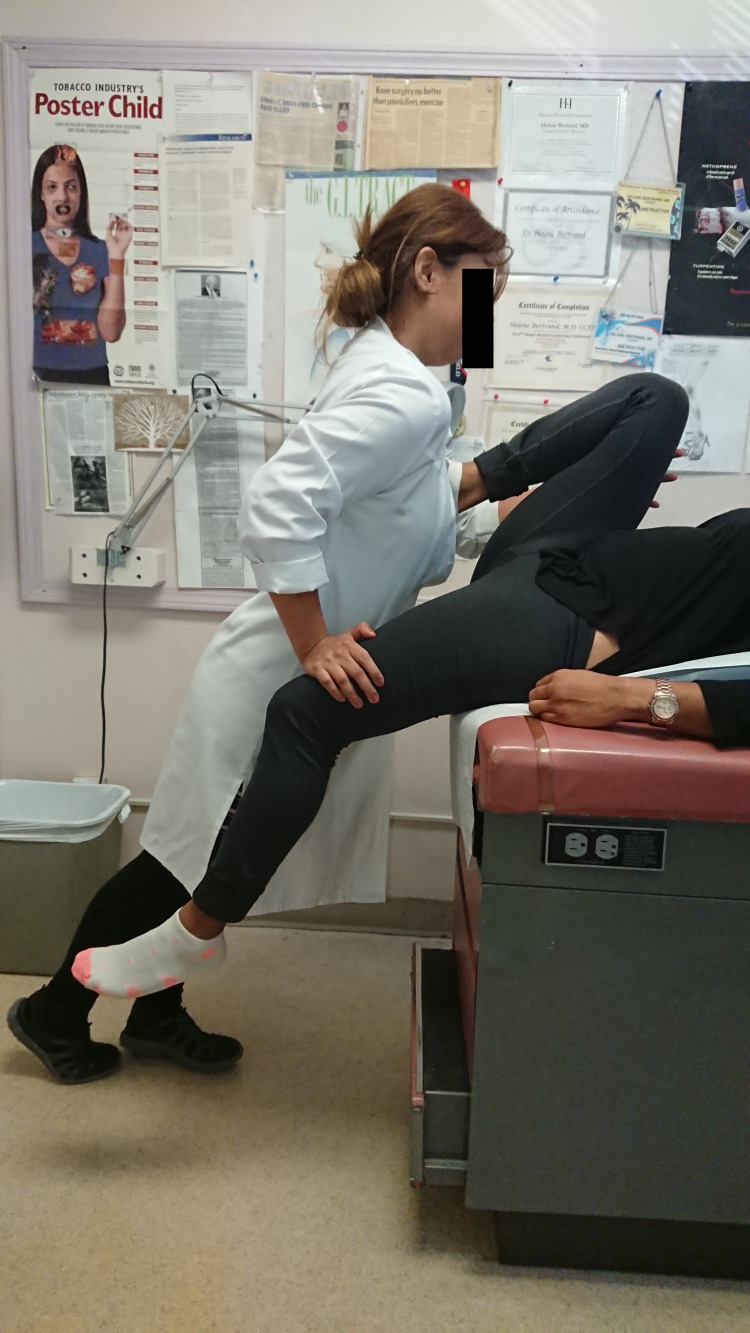
SIFFT-E treating a right anterior, left posterior sacroiliac torsion in dorsal decubitus The patient lies in dorsal decubitus with their buttock on the edge of the examination table, right thigh flexed, and left thigh extended. The patient's right foot is placed against a cushion on the examiner's sternum, and the examiner leans forward hard to force the right thigh against the right ASIS for a right anterior SI torsion. The examiner presses down hard on the left thigh to hyperextend it for a left posterior SI torsion. If only one side is affected, the nonpainful side is simply held in place. The position is held for a full two minutes. Three of the 62 participants needed this treatment due to limited range of motion or their enlarged abdomen interfering with hip flexion in the stretch exercise. SIFFT, Sacroiliac forward flexion test; SIFFT-E, SIFFT-guided leveling exercise; SI, sacroiliac; ASIS, anterior superior iliac spine.

**Figure 6:**
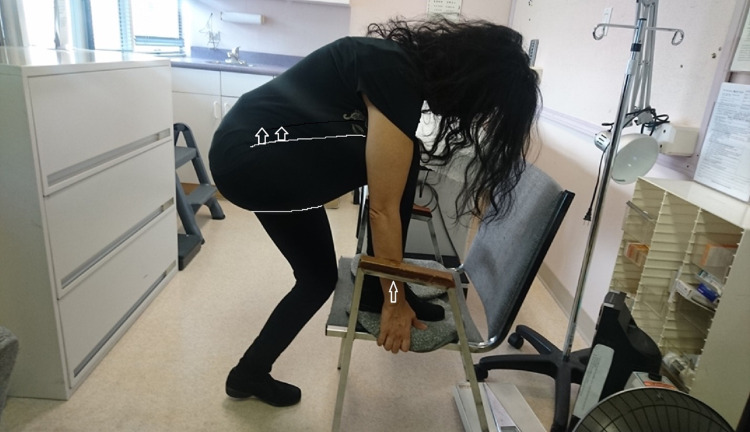
SIFFT-E treating a right anterior sacroiliac torsion: The right thigh pushes the right ASIS backward Place the right foot on the seat of the chair with the hands holding the seat on either side of the foot. The left knee touches the front of the seat. Lean back. Pull up hard with both hands on the seat of the chair and hold for a full two minutes. Six of the 62 participants did the chair exercise. It indicated when the only tender PSIS is anteriorly displaced (higher than the other PSIS) and the arms are sufficiently strong to hold a two-minute pull. SIFFT, Sacroiliac forward flexion test; SIFFT-E, SIFFT-guided leveling exercise; ASIS, anterior superior iliac spine; PSIS, posterior superior iliac spine.

The SIFFT-E used the ipsilateral thigh to correct sacroiliac malrotation (See Figures 4-6). A painful PSIS that was higher than the other was diagnosed as an anteriorly displaced innominate bone. The corrective exercise used the thigh pushing against the anterior superior iliac spine (ASIS) to force it posteriorly. If the painful PSIS was lower than the other, its innominate bone was diagnosed as posteriorly displaced, in which case hyperextension of the thigh used the sartorius on the ASIS and the rectus femoris on the anterior inferior iliac spine (AIIS) to pull the posteriorly displaced innominate bone forward [19]. Each position was held for two minutes. Depending on their ability to perform the corrective exercise, participants in group 1 were given one of three leveling exercises (SIFFT-E). The stretch exercise was given to those with normal mobility (Figure 4).

Inspect for those with obesity or limited range of motion (they could not place their hands on the floor on either side of their foot), so the exercise can be done in dorsal decubitus with the help of an assistant (Figure 5).

If the only tender joint was displaced anteriorly and the patient preferred to stand, the chair exercise was taught (Figure 6). The corrective exercise uses force on the painful side. The asymptomatic side is not strained (see flowchart). Similar exercises were described by Horton in a case report [19].

**Figure 7:**
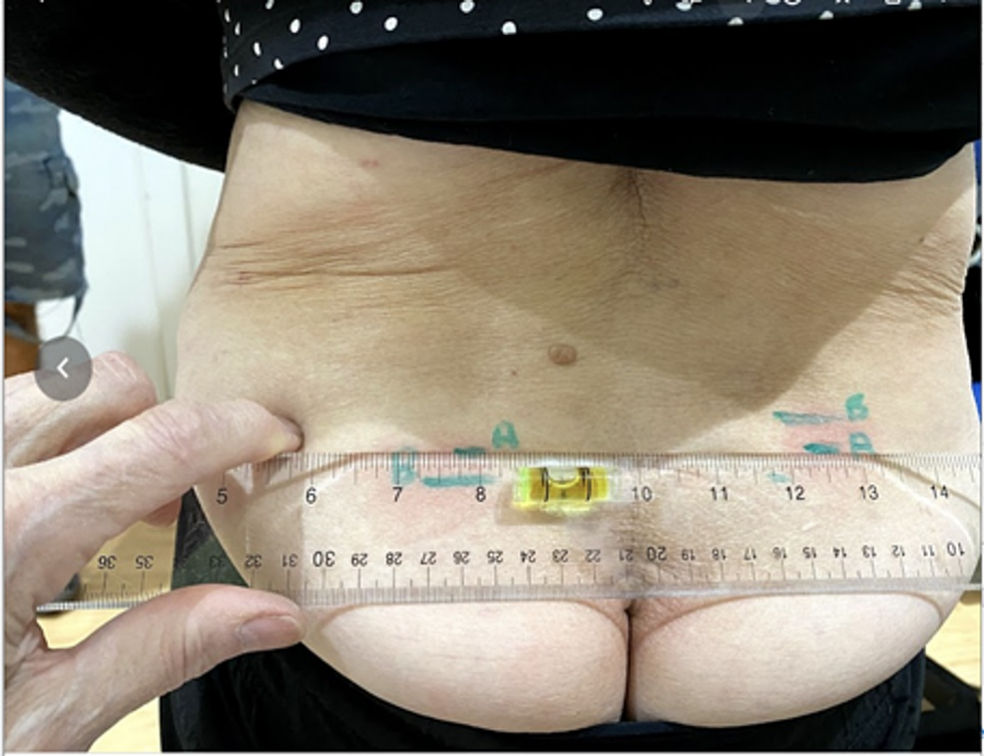
Repeat SIFFT test done after the SIFFT-E After the two-minute SIFFT-E is done, the SIFFT is repeated, and the location of the PSISs is marked with the letter A for after. The level is used to measure their alignment. As a rule, when the PSISs are level, the back pain and limitation of function are relieved. In this participant who had pain on both sides, the higher PSIS on the right indicates a right anterior torsion of the innominate bone on the sacrum, while the lower PSIS on the left points to a left posterior torsion of the innominate bone on the sacrum. SIFFT, Sacroiliac forward flexion test; SIFFT-E, SIFFT-guided leveling exercise; PSIS, posterior superior iliac spine.

Following this, the SIFFT was repeated. The letter A (indicating “after”) was written beside the mark corresponding to each PSIS (Figure 7). If the PSISs were still not level and pain persisted, the leveling exercise was repeated.

Group 1 participants were referred to an instructional video on how to diagnose displaced sacroiliac joints and how to treat them. Participants used the SIFFT-E on their own as needed for pain relief for one month, following which they were fitted with the pelvic stabilizer belt to use as needed.

Group 2: Pelvic Stabilizer Belt

To support sacroiliac stability and potentially improve asymmetry, function, and pain [16,17,20,21], participants in group 2 were fitted with a pelvic stabilizer belt (acknowledgments). They were taught how to locate their ASISs and told to always place the belt below these ASISs as the iliac bones flare outward and a belt squeezing the iliac crests may open the sacroiliac joints, making them more unstable. Participants wore the belt as needed whenever doing activities known to aggravate their LBP for one month, following which they were taught the SIFFT-E and allowed to continue using their belt as needed. Group 2 participants were referred to a video instructing them on how to apply and wear a pelvic stabilization belt.

The examination technique, the corrective exercises, and how to don a pelvic stabilizer belt are presented in Video 1.

**Video 1:** How to diagnose and treat low back pain from sacroiliac joint displacement

Group 3: Observation

Participants in group 3 were instructed to continue with their current treatments (physiotherapy, chiropractic manipulation, massage therapy, acupuncture, yoga, core exercises, medications, cannabis, and narcotics) and were told to return in one month. When they returned, they were taught the SIFFT-E and then fitted with the sacroiliac support belt to be used as needed.

Participants were not asked to stop the usual care during the study but were discouraged from beginning additional treatments. All participants were instructed to return to the clinic for their last assessment one month after using both treatments as needed. This was the endpoint of data collection.

Outcomes

Our primary outcome was the Oswestry disability index (ODI), a measure of back function which has a range of minimal clinically important differences (MCIDs), depending on the condition studied [22], and 11 after spinal surgery [23].

Secondary outcomes included the SIFFT (difference in millimeters between right and left PSIS levels) and the BPI severity score (MCID 2 in LBP) [24]. Data for ODI, SIFFT, and BPI were collected at baseline and one and two months later. 

Other secondary outcome measures included the collection of a 0-10 numeric rating scale (NRS) pain score at each visit, before and immediately after the SIFFT exercise and/or the pelvic stabilizer belt fitting. After all the participants had used both exercise and belt for one month, their preference for the SIFFT-E, the pelvic stabilizer belt, both, or neither was recorded, Adverse events were recorded by the researchers if they occurred during a visit or by the participants on their return visit questionnaire.

Sample Size Determination

No information on the expected standard deviation of the effect of SIFFT-E on the ODI was available. We chose a sample size of 20 in each group. This was an informal estimate based on the quality assurance data gathered in the primary investigator’s clinic on the effect of SIFFT-E compared to usual care on pain, a different primary variable. In addition, this sample size is in the range recommended for a pilot study with a relatively small effect size.

Data and Statistical Analysis

At all visits, the 0-10 NRS for pain was recorded immediately before and after using the SIFFT-E and/or the pelvic stabilizer belt. At the end of the first month, those using SIFFT-E were compared with belt only and usual care participants on the change in ODI, the SIFFT, and the BPI pain severity from baseline to one month. 

After all the participants used both SIFFT-E and the pelvic stabilizer belt for one month, their ODI, SIFFT, and BPI pain severity were compared to baseline. The statistical program used was PASW Statistics 18, Release 18.0.0 (IBM Corp., Armonk, New York). The trial was registered at www.clinicaltrials.gov (NCT03888235).

If the data sets were not normally distributed using the Shapiro-Wilk analysis for normal distribution, the Kruskal-Wallis one-way analysis of variance for two or more independent samples (groups) was used to test for a significant between-group difference across all groups at P < 0.05. If the P-value was significant, a Mann-Whitney U test was applied to each pair to determine for which pairs the between-group difference was significant. Because three groups were evaluated pair by pair, the required P-value was < 0.0167 by Bonferroni correction. To compare groups for the number of participants achieving clinically significant (MCID level) improvements in ODI from zero to one month, Fisher's exact test was performed as a post-hoc analysis, which does not require a Bonferroni correction. Time 0-to-2-month data were analyzed similarly. If a significant difference across all three groups was not identified, group data were combined for analysis. There was no data-monitoring committee. The protocol and the results were reported in clinical trials.gov (NCT03888235).

## Results

Participants were first seen between November 28, 2019, and August 31, 2020. Their baseline characteristics did not differ significantly (Table 1).

**Table 1:** Baseline analysis by group ODI, Oswestry disability index; SI asymmetry (mm): distance in millimeters between the PSIS levels; BPI, brief pain inventory; PSIS, posterior superior iliac spine.

Variable		SI Exercise, n = 21	SI Belt, n = 21	Usual Care, n = 20
Age	Mean (SD)	53 (16)	51 (12)	54 (13)
Age	Median (IQR)	50 (28)	49 (13)	55 (12)
Race	White	18 (86%)	14 (67%)	19 (95%)
Race	African-American	1 (5%)	0	0
Race	Other	2 (9%)	7 (33%)	1 (5%)
Gender	Female	15 (71%)	13 (62%)	10 (50%)
Pain duration (months)	Mean (SD)	90 (121)	74 (86)	119 (148)
Pain duration (months)	Median (IQR)	36 (108)	60 (113)	60 (144)
Baseline ODI	Mean (SD)	40 (15.6)	36 (17.4)	38 (16.4)
Baseline ODI	Median (IQR)	40 (25)	38 (25)	32 (13)
SI asymmetry (mm)	Mean (SD)	15 (6.9)	14 (6.7)	13 (6.1)
SI asymmetry (mm)	Median (IQR)	15 (9)	12 (6)	10 (9)
Baseline BPI pain	Mean (SD)	6.0 (1.8)	5.2 (1.9)	6.1 (1.7)
Baseline BPI pain	Median (IQR)	6.5 (3.0)	5.8 (2.8)	5.9 (2.8)
Gender	Female	15 (71%)	13 (62%)	10 (50%)

**Figure 8:**
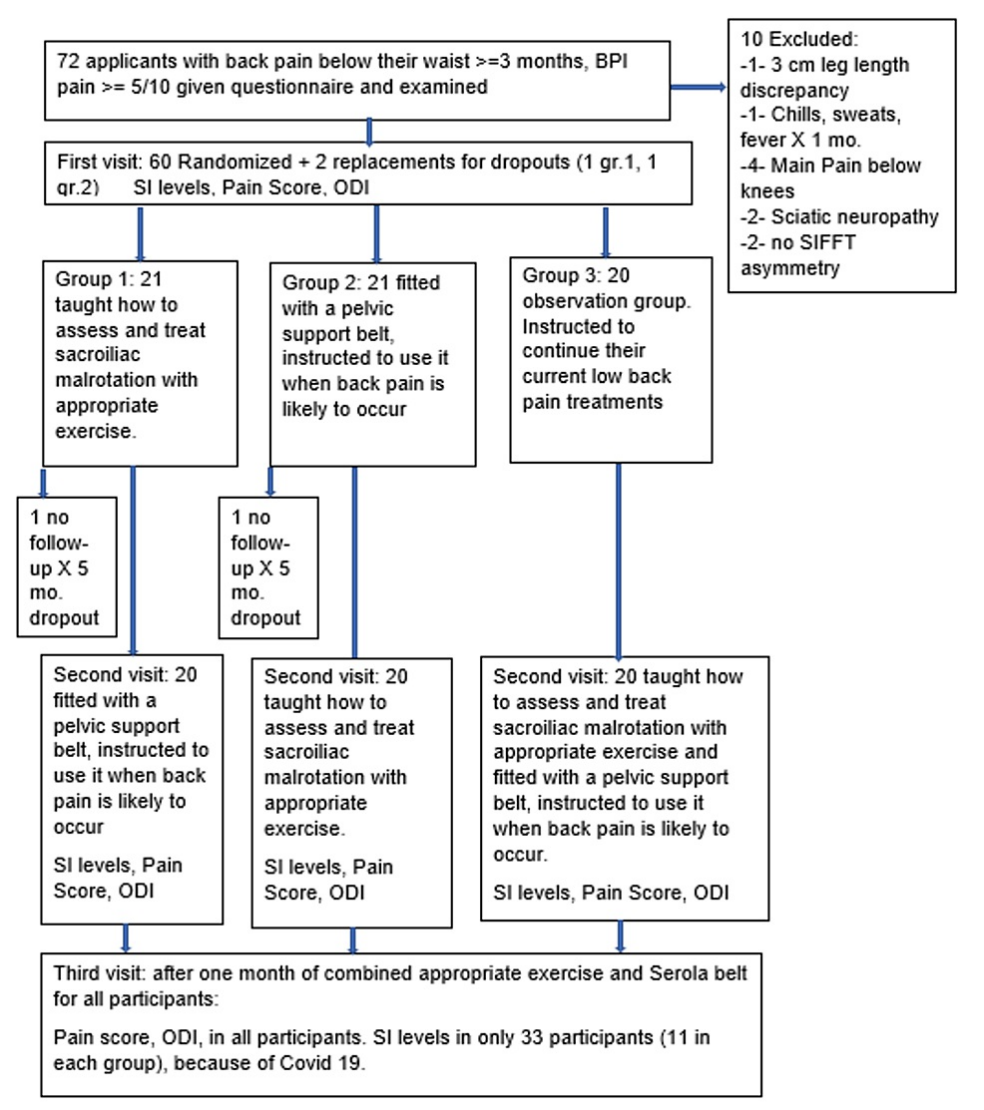
Trial profile for low back pain study ODI, Oswestry disability index; SI, sacroiliac.

There were 75 callers to the office with LBP > three months, but three said they could not return for follow-up, so 72 were given an assessment appointment (Figure 8). A SIFFT asymmetry of 0.5 cm or more was observed in 86% (62/72) of consecutive outpatients with a complaint of LBP who then became eligible for the study. All 62 accepted an offer of study participation. Of those, 10 required lifts to correct leg length discrepancy. Twenty-one participants were assigned to group 1 with one dropout, 21 to group 2 with one dropout, and 20 to group 3. After one month in their assigned group, all 60 remaining participants were given both the exercise and the belt. They reported back after using them for one month and filled out their two-month questionnaire.

Our middle-aged (53 ± 13 years) participants were predominantly females (38/62; 61%) with chronic pain (90 ± 120 months with only five participants reporting pain < six months). They had moderately severe pain (BPI 5.8 ± 1.8), high-moderate ODI levels (38 ± 16.3), and substantial SI asymmetry (14.0 ± 6.5 mm). Fifty-two of 62 participants reported they had pain every day, six averaged 2.2 painful days per week, and four suffered an average of 3.3 days per month.

Therapies received previously by the 62 participants, similarly distributed between groups, included physical therapy in 50, massage therapy in 45, chiropractic manipulation in 44, core exercises in 39, acupuncture in 35, and yoga in 29.

The group that was given a leveling exercise appropriate for the direction of SI asymmetry (SIFFT-E) experienced significantly more improvement in ODI than usual care participants (12.5 ± 14.8 vs. -3.4 ± 14.9 points; mean difference 15.9 points [CI = 6.7- 25.0]; P = 0.002) (Table 2). The percentage of participants in usual care and SIFFT-E that did not achieve a ≥ 11 improvement in ODI was 0.90 and 0.57, respectively, for an absolute risk reduction of 0.33 and a number needed to treat (NNT) of 1/0.33 or 3.0.

**Table 2:** Change in Oswestry disability index (ODI) before and after the use of exercise and belt ^1^ Exercise outperformed the usual care for overall ODI improvement (12.5 ± 14.8 vs. -3.4 ± 14.9 points; P = 0.002). Exercise nearly outperformed the pelvic stabilizer belt for overall ODI improvement (12.5 ± 14.8 vs. 4.3 ± 15.1 points; P = 0.023 with Bonferroni correction; P-value = 0.017). However, the Fisher's exact test revealed that significantly more SIFFT-E participants achieved an ODI improvement ≥ 11 compared to the belt-use participants (9/21 vs. 3/21; P = 0.043. Note that Bonferroni correction is not required for Fisher's exact test. When used alone, the belt did not outperform usual care after Bonferroni correction for ODI (P = 0.31). ^2 ^Each group received the same treatment from one to two months (SI exercise and SI belt). ^3 ^No significant between-group differences were seen upon non-parametric (Kruskal-Wallace) testing (P = 0.81). ODI, Oswestry disability index.

		SI Exercise, n = 21	SI Belt, n = 21	Usual Care, n = 20
ODI time 0 Baseline	Mean (SD)	41 (16)	36 (17)	38 (16)
	Median (IQR)	40 (25)	38 (25)	32 (13)
ODI 1 month	Mean (SD)	28 (18)	32 (16)	42 (17)
	Median (IQR)	28 (35)	32 (30)	39 (26)
ODI 2 months	Mean (SD)	30 (16)	23 (18)	26 (13)
	Median (IQR)	29 (25)	18 (29)	25 (19)
ODI 0-1 month	Mean (SD)	13 (15)^1^	4 (15)^1^	-3 (14)^1^
	Median (IQR)	8 (18)	2 (13)	-2 (13)
ODI 0-2 months^2^	Mean (SD)	11 (11)^3^	13 (22)^3^	13 (22)^3^
	Median (IQR)	8 (13)	12 (30)	0 (72)

The SIFFT-E participants did not outperform the belt-use participants for overall ODI improvement: statistical correction for multiple groups (12.5 ± 14.8 vs. 4.3 ± 15.1 points; mean difference 8.2 points [CI -1.1-17.5]; P = 0.023 with the Bonferroni correction P-value of 0.017). However, significantly more SIFFT-E participants achieved an ODI improvement ≥ 11 compared to the belt-use participants (9/21 vs. 3/21; P = 0.043 by Fisher’s exact test).

SIFFT-E participants also showed more mean reduction in asymmetry (7.3 ± 9 mm vs. -1 ± 10 mm; mean difference 8 mm [CI = 1.3-8.8 mm; P = 0.005]) than the usual care group. When used alone, the belt did not outperform the usual care after Bonferroni correction for either ODI (P = 0.31) or SI asymmetry (P = 0.029 with Bonferroni correction P-value of 0.017). Because of COVID, only 11 participants in each group (a total of 33/62) were able to be examined for SI asymmetry in the clinic (Table 3). They averaged a reduction in SIFFT of 8.6 ± 8.6 mm, P < 0.001.

**Table 3:** Change sacroiliac joint (SI) asymmetry before and after the use of exercise and belt ^1 ^Exercise outperformed the usual care for improvement (reduction) in SI asymmetry (7 ± 9 mm vs. -1 ± 10 mm; P = 0.005) but did not outperform the belt-only group (7 ± 9 mm vs 5 ± 7 mm; P = 0.21). ^2 ^The belt-only group did not outperform the usual care (5 ± 7 mm vs -1 ± 10 mm; P = 0.029, but Bonferroni corrected alpha was 0.0167). ^3^ Each group received the same treatment from one to two months (SI exercise and SI belt), and n = 11 for each group for SI asymmetry analysis at two months due to limited in-person follow-up (COVID-19 related). ^4 ^No significant between-group differences were seen upon non-parametric (Kruskal-Wallace) testing (P = 0.42). When used alone, the belt did not outperform usual care after Bonferroni correction for either ODI (P = 0.31) or SI asymmetry (P = 0.029 with Bonferroni correction P-value of 0.017).

		SI Exercise, n = 21	SI Belt, n = 21	Usual Care, n = 20
SI asymmetry baseline (mm)	Mean (SD)	15 (7)	14 (7)	13 (6)
	Median (IQR)	15 (9)	12 (6)	10 (9)
SI asymmetry 1 month (mm)	Mean (SD)	8 (7)	9 (5)	14 (9)
	Median (IQR)	5 (14)	10 (8)	10 (13)
SI asymmetry 2 months^2 ^(mm)	Mean (SD)	4 (5)	6 (5)	6 (10)
	Median (IQR)	2 (5)	8 (10)	0 (10)
SI asymmetry 0–1 month (mm)	Mean (SD)	7 (9)^1^	5 (7)^1^	-1 (10)^1^
	Median (IQR)	8 (13)	5 (8)	0 (10)
SI asymmetry 0–2 months^3^ (mm)	Mean (SD)	12 (8)^3^	7 (5)^3^	7 (12)^3^
	Median (IQR)	10 (10)	10 (8)	10 (20)

There were no significant between-group differences for pain at one month. Improvements in BPI pain from zero to one month trended better for exercise than usual care but did not reach clinical significance. (1.3 ± 2.3 vs. 0.3 ± 1.4; P = 0.053).

After all 62 participants (baseline values for the two dropouts was carried forward) used SIFFT-E and belt as needed for one month, improvements were statistically and clinically significant for both functions (ODI; 13 ± 22; P < 0.001) and BPI pain (2.2 ± 2.2; P < 0.001). Tables 2, 4 show that the ODI and BPI pain improvements were ≥ MCID for ODI of 11 and BPI pain of 2, (25) in each of the three groups.

**Table 4:** Change in brief pain inventory (BPI) pain score before and after the exercise and belt ^1^ No significant between-group differences were seen upon non-parametric (Kruskal-Wallace) testing (P = 0.17). ^2 ^Each group received the same treatment from one to two months (SI exercise and SI belt). ^3 ^No significant between-group differences were seen upon non-parametric (Kruskal-Wallace) testing (P = 0.93).

		SI Exercise, n = 21	SI Belt, n = 21	Usual Care, n = 20
BPI pain score baseline	Mean (SD)	6.0 (1.8)	5.3 (1.9)	6.2 (1.7)
	Median (IQR)	6.5 (3.0)	5.8 (2.8)	All (0.8)
BPI pain score 1 month	Mean (SD)	4.7 (2.5)	4.1 (2.4)	5.9 (2.3)
	Median (IQR)	4.3 (4.3)	4.0 (3.1)	6.0 (4.4)
BPI pain score 2 months	Mean (SD)	4.0 (2.4)	2.9 (2.1)	3.8 (2.2)
	Median (IQR)	4.0 (3.8)	2.5 (3.6)	3.6 (3.3)
BPI pain score 0-1 month	Mean (SD)	1.3 (2.3)^1^	1.2 (2.1)^1^	0.3 (1.4)^1^
	Median (IQR)	1.3 (2.0)	1.0 (3.1)	0.3 (2.4)
BPI pain score 0-2 months^2^	Mean (SD)	2.0 (1.9)^3^	2.3 (2.2)^3^	2.3 (2.5)^3^
	Median (IQR)	1.8 (3.0)	1.8 (3.5)	1.9 (3.4)

**Figure 9:**
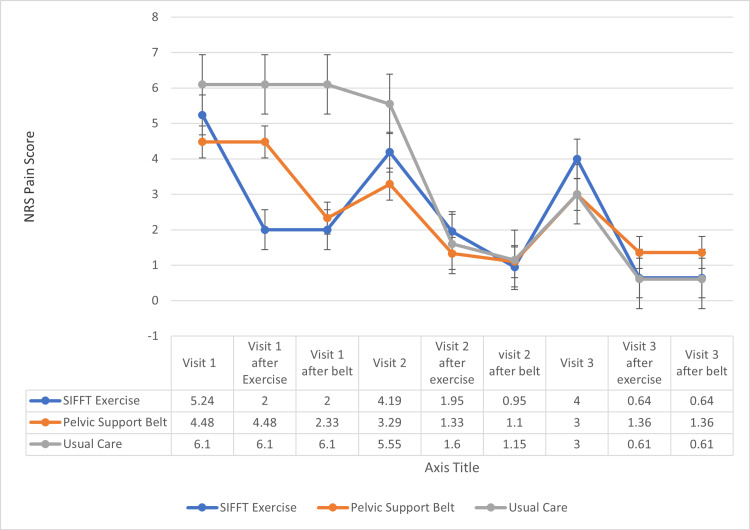
NRS (0-10) pain severity response to exercise or belt Changes with SIFFT-E at time 0 (group 1, n = 21) (5.2 ± 2.5 to 2.0 ± 2.4 (62%); change score 3.2 ± 2.6; CI = 2.0-4.4; P < 0.0001). As those assigned to SIFFT-E did not receive the belt, their pain score was carried forward for “visit 1 after belt”. Changes with SI stabilization belt at time 0 (group 2, n = 21). As they did not receive the SIFFT-E, their visit 1 pain score was carried forward for “visit 1 after exercise” (4.5 ± 2.3 to 2.3 ± 2.2 (49%); change score 2.1 ± 1.8; CI = 1.4-2.9; P < 0.001). As those in group 3 received no treatment in visit 1, the visit 1 pain scores were carried forward to “visit 1 after exercise” and to “visit 1 after belt”. Changes with SIFFT-E at 1 month (n = 62) (4.3 ± 2.7 to 1.6 ± 2.0 (63%); change score 2.7 ± 2.1; CI = 2.2-3.2; P < 0.0001). Changes with SI stabilization belt (after SIFFT-E) at 1 month (n = 62) (1.6 ± 2.0 to 1.1 ± 1.5 (31%); change score 0.6 ± 1.0; CI = 0.3-0.8; P < 0.001). NRS, Numeric rating scale; SIFFT, sacroiliac-leveling exercise; SIFFT-E, SIFFT-guided leveling exercise; SI, sacroiliac.

An immediate reduction of pain in 90% of the participants was observed after the performance of the SIFFT-E at the baseline (19/21) and one-month (54/60) visits. Of those who received the belt at the first visit, 86% found relief (18/21), and 95% of those received the belt at the second visit (57/60) when they were fitted with the belt after being taught and having performed the SIFFT-E (Figure 9).

The number of times the SIFFT-E was repeated was only recorded by the primary investigator, accounting for 40 of the 131 visits. During those 40 visits, only one patient needed to be treated a second time. That patient was hypermobile, and her pelvic asymmetry switched directions with the two-minute SIFFT-E, requiring the opposite exercise for only one minute. Following this, her PSISs were level, and she was pain-free, an example of the importance of using the SIFFT to determine the correct SIFFT-E.

For 40 participants, asymmetry determinations (side and direction of asymmetry) were made by different observers on the same participant at two different points in time. The same side/direction pair was recorded in 30/40 participants. Of the 20 participants measured only by one observer at two different points in time, the side/direction pair recorded was consistent in 18/20. Given four possible choices for side/direction pairs from a visit to visit, these results suggest at least moderate intra- and interobserver reliability. However, this data more accurately reflects the stability of the direction of asymmetry rather than confirming reliability.

Among the 60 participants who used both SIFFT-E and belt, each as needed, more participants preferred the exercise alone 21/60 (35%) than belt alone 8/60 (13.3%), but most preferred using both 29/60 (49%). Two (3.3%) preferred neither. Five participants (8%) experienced mild side effects from the exercise. One of the five (1.6%) was hypermobile (Beighton score = 5) and overcorrected her sacroiliac malrotation. Three felt some leg pain, and one had difficulty bending over to the garden. Thirteen (21%) had side effects from the belt. In seven, the belt caused rubbing and discomfort, three had skin irritation, and three felt the belt restricted their movements. There were no serious side effects.

## Discussion

Our observation that 86% of consecutive patients seen for chronic LBP had SI asymmetry of more than 5 mm suggests that SI asymmetry is a very common examination finding in the general population with LBP. Study participants doing the SIFFT-E had a greater reduction in functional limitations and SI asymmetry than those in the usual care group. Those receiving the pelvic stabilization belt had intermediate results. The improvement in function, asymmetry, and pain in all participants was clinically and statistically significant for one month after they received both SIFFT-E and SI belts and used each as needed. In patients with asymmetry, we demonstrated prompt and significant reduction of pain immediately after the SIFFT-E was completed.

Non-radicular LBP is still considered to be nonspecific with a variety of often expensive treatments advocated and limited evidence of efficacy [25-28]. This study also suggests that SI asymmetry is measurable and correctable with the use of a rapidly learned self-treatment, which provides significant symptom reduction and improvement in function. Practical application of the approach taken in this study would require practitioners to simply test for and correct leg length asymmetry and then perform the SIFFT with manual palpation or ultrasound to locate the levels of the inferior edge of the PSISs. A ruler, preferably clear, to which a small level has been glued (Figures 2, 7), and a skin marker are the only instruments needed for this test. In our experience, the SIFFT and SIFFT-E can be learned in one to two hours with three to four patients, and the assessment with initial exercise completion can be completed within 10 minutes.

Although some will require repetition of the exercise, as did one participant in this study, the majority (90%) of participants had an immediate reduction in pain following the SIFFT-E. This suggests that the SIFFT followed by the SIFFT-E may serve as a "diagnostic test" for sacroiliac asymmetry as a cause of LBP, postponing or reducing the need for expensive medical imaging, prescription medications, or referrals for more specialized and costly care. It allows patients to immediately relieve and control their own LBP.

The probable cause of SI asymmetry is weakness in the ligaments stabilizing the SI joints. This could be from an accidental sprain or tear, the effect of relaxin in pregnancy, the weak collagen in hypermobility, or the loss of collagen from old age. These joints absorb the shock of every step and are strained with prolonged sitting, lifting, or bending forward, which explains why they are surrounded by more ligaments than any other joint in the body [21]. When the ligaments are weak, any one of these activities can displace the innominate bone, causing painful SI asymmetry, which may be why half of the participants preferred a combination of the SIFFT-E and the pelvic support belt to be used as needed.

Strengths of this study include the following: It provides preliminary data suggesting that (1) sacroiliac asymmetry can be diagnosed with simple tools available to the general practitioner, (2) the examination is sufficiently accurate to provide guidance for a corrective maneuver that patients can use as needed to treat their own pain, (3) the exercise may infrequently require repetition, (4) self-treatment may result in a clinically important benefit, and (5) prompt pain relief may be frequent enough to serve as a diagnostic tool, limiting the need for sophisticated or expensive testing and/or injections.

Limitations of this study include the short study period. The individual treatments were only assessed over one month and the combined use of the corrective exercise and pelvic support belt was assessed for an additional month. The study was also small as each group only had 20 participants and it was carried out in one site by three different physicians. Others might have more difficulty exactly locating the PSIS levels, particularly in patients with obesity, suggesting the need for the use of ultrasound for measurement. In this study, the palpation of the anatomical area was not confirmed by an ultrasound study, and the improvements in the sacroiliac joint may be linked to anatomical or neurophysiological adaptations not still completely elucidated.

Due to block size limitation to three, the ability to predict the next group would have been present with the close attention of the treating physician. However, the allocation would not have been affected since data were collected prior to group assignment and this is an open-label study. The replacement of two participants was problematic as these two would not have been randomized per protocol. They both were assigned to what would be recognized as active treatment, but it diminishes the quality of otherwise randomization and random allocation.

This study was limited by a lack of formal inter- and intraobserver reliability testing. Therefore, whether the relatively high degree of intraobserver correlation seen by Levangie on measurement of floor to PSIS height will be demonstrated with similar formal SIFFT evaluation remains to be determined [18]. Irrespective of inter- and intraobserver reliability of the SIFFT, unstable innominate bones are susceptible to change their direction on the sacrum indicating the need to repeat the SIFFT at each visit.

## Conclusions

In patients presenting with LBP, locating the PSISs and comparing their levels (SIFFT) usually lead to a diagnosis of sacroiliac malrotation that may be correctable with a two-minute exercise, resulting in significant pain relief and functional improvement. A study comparing the accuracy of palpation to that of ultrasound in finding the PSIS levels is needed. The current study needs to be replicated with more participants in multiple sites for a longer period. If replicated successfully, this simple, non-pharmacologic approach may be a useful, low-cost supplement to our current paradigm of LBP assessment and therapeutics.

## Figures and Tables

**Figure 10:**
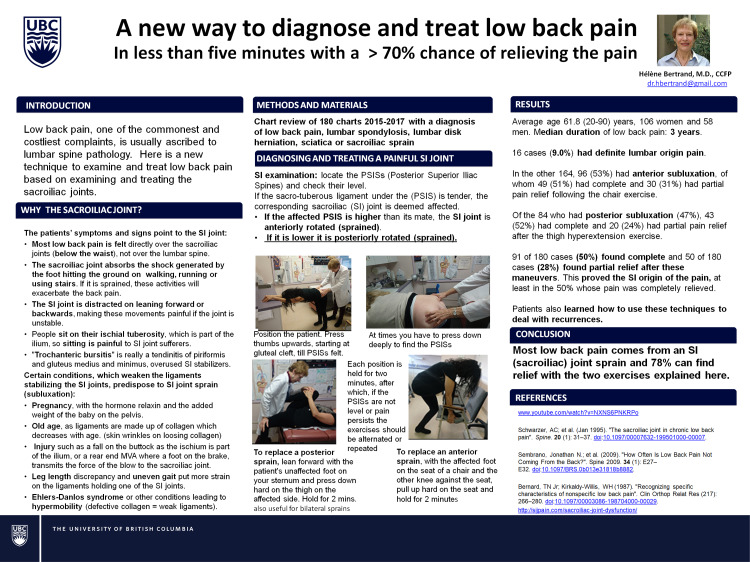
A new way to diagnose and treat low back pain in <5 minutes with >70% chance of relieving the pain Poster presentation #119, Canadian pain Society meeting May 24, 2018. A total of 180 charts (2015–2017) were reviewed. If the sacrotuberous ligament under a PSIS was tender, the corresponding SI joint was deemed affected. If the affected PSIS was higher than its mate, the SI joint was anteriorly subluxed. If it was lower, it was posteriorly subluxed. Anterior subluxation was corrected by jamming the thigh on the affected side against the anterior superior iliac spine (ASIS) using a chair. Posterior subluxation correction involved hyperextending the thigh on the affected side. Each exercise was held for two minutes. The chair exercise was strenuous, and the examining table treatment required an assistant. This prompted the development of the stretch exercise to allow low back pain sufferers a way to self-relieve their pain at all times. Exact measurement of the distance between the right and left PSIS levels was not taken. To measure the patients’ exercise effectiveness and their progress in leveling the PSISs, the SIFFT was developed. SIFFT, Sacroiliac-leveling exercise; PSIS, posterior superior iliac spine.

## Appendices

Appendix A

## References

[1] Kim LH, Vail D, Azad TD (2019). Expenditures and health care utilization among adults with newly diagnosed low back and lower extremity pain. JAMA Netw Open.

[2] Fatoye F, Gebrye T, Odeyemi I (2019). Real-world incidence and prevalence of low back pain using routinely collected data. Rheumatol Int.

[3] Lin CW, Haas M, Maher CG, Machado LA, van Tulder MW (2011). Cost-effectiveness of guideline-endorsed treatments for low back pain: a systematic review. Eur Spine J.

[4] Ferguson SA, Merryweather A, Thiese MS, Hegmann KT, Lu ML, Kapellusch JM, Marras WS (2019). Prevalence of low back pain, seeking medical care, and lost time due to low back pain among manual material handling workers in the United States. BMC Musculoskelet Disord.

[5] Deyo RA, Weinstein JN (2001). Low back pain. N Engl J Med.

[6] Amundsen PA, Evans DW, Rajendran D (2018). Inclusion and exclusion criteria used in non-specific low back pain trials: a review of randomised controlled trials published between 2006 and 2012. BMC Musculoskelet Disord.

[7] Rahyussalim AJ, Zufar ML, Kurniawati T (2020). Significance of the association between disc degeneration changes on imaging and low back pain: a review article. Asian Spine J.

[8] Jarvik JG, Deyo RA (2002). Diagnostic evaluation of low back pain with emphasis on imaging. Ann Intern Med.

[9] Babińska A, Wawrzynek W, Czech E, Skupiński J, Szczygieł J, Łabuz-Roszak B (2019). No association between MRI changes in the lumbar spine and intensity of pain, quality of life, depressive and anxiety symptoms in patients with low back pain. Neurol Neurochir Pol.

[10] Potter NA, Rothstein JM (1985). Intertester reliability for selected clinical tests of the sacroiliac joint. Phys Ther.

[11] Szadek KM, van der Wurff P, van Tulder MW, Zuurmond WW, Perez RS (2009). Diagnostic validity of criteria for sacroiliac joint pain: a systematic review. J Pain.

[12] Kolber MR, Ton J, Thomas B (2021). PEER systematic review of randomized controlled trials: management of chronic low back pain in primary care. Can Fam Physician.

[13] Enke O, New HA, New CH (2018). Anticonvulsants in the treatment of low back pain and lumbar radicular pain: a systematic review and meta-analysis. CMAJ.

[14] Ferreira GE, McLachlan AJ, Lin CC, Zadro JR, Abdel-Shaheed C, O'Keeffe M, Maher CG (2021). Efficacy and safety of antidepressants for the treatment of back pain and osteoarthritis: systematic review and meta-analysis. BMJ.

[15] Shadmehr A, Jafarian Z, Tavakol K, Talebian S (2013). Effect of pelvic compression on the stability of pelvis and relief of sacroiliac joint pain in women: A case series. J Musculoskelet Pain.

[16] Mens JM, Damen L, Snijders CJ, Stam HJ (2006). The mechanical effect of a pelvic belt in patients with pregnancy-related pelvic pain. Clin Biomech (Bristol, Avon).

[17] Hammer N, Möbius R, Schleifenbaum S (2015). Pelvic belt effects on health outcomes and functional parameters of patients with sacroiliac joint pain. PLoS One.

[18] Levangie PK (1999). Four clinical tests of sacroiliac joint dysfunction: the association of test results with innominate torsion among patients with and without low back pain. Phys Ther.

[19] Horton SJ, Franz A (2007). Mechanical diagnosis and therapy approach to assessment and treatment of derangement of the sacro-iliac joint. Man Ther.

[20] Pool-Goudzwaard AL, Vleeming A, Stoeckart R, Snijders CJ, Mens JM (1998). Insufficient lumbopelvic stability: a clinical, anatomical and biomechanical approach to 'a-specific' low back pain. Man Ther.

[21] Enix DE, Mayer JM (2019). Sacroiliac joint hypermobility biomechanics and what it means for health care providers and patients. PM R.

[22] Schwind J, Learman K, O'Halloran B, Showalter C, Cook C (2013). Different minimally important clinical difference (MCID) scores lead to different clinical prediction rules for the Oswestry disability index for the same sample of patients. J Man Manip Ther.

[23] Yoshida G, Hasegawa T, Yamato Y (2019). Minimum clinically important differences in Oswestry disability index domains and their impact on adult spinal deformity surgery. Asian Spine J.

[24] Hägg O, Fritzell P, Nordwall A (2003). The clinical importance of changes in outcome scores after treatment for chronic low back pain. Eur Spine J.

[25] Cherkin DC, Herman PM (2018). Cognitive and mind-body therapies for chronic low back pain and neck pain: effectiveness and value. JAMA Intern Med.

[26] Chou R, Qaseem A, Snow V, Casey D, Cross JT Jr, Shekelle P, Owens DK (2007). Diagnosis and treatment of low back pain: a joint clinical practice guideline from the American College of Physicians and the American Pain Society. Ann Intern Med.

[27] Buchbinder R, van Tulder M, Öberg B, Costa LM, Woolf A, Schoene M, Croft P (2018). Low back pain: a call for action. Lancet.

[28] Skelly AC, Chou R, Dettori JR (2020). Noninvasive Nonpharmacological Treatment for Chronic Pain: A Systematic Review Update. Rockville (MD): Agency for Healthcare Research and Quality (US). AHRQ Comparative Effectiveness Reviews.

